# A deep dive into artificial intelligence with enhanced optimization-based security breach detection in internet of health things enabled smart city environment

**DOI:** 10.1038/s41598-025-05850-z

**Published:** 2025-07-02

**Authors:** S Jayanthi, Sodagudi Suhasini, N. Sharmili, E. Laxmi Lydia, V. Shwetha, Bibhuti Bhusan Dash, Mrinal Bachute

**Affiliations:** 1 Department of Artificial Intelligence & Data Science, Faculty of Science and Technology (IcfaiTech), The ICFAI Foundation for Higher Education (IFHE), Hyderabad, Telangana 01 503 India; 2 Department of Information Technology, Siddhartha Academy of Higher Education, (Deemed to Be University), Kanuru, Vijayawada-07, AP India; 3 Computer Science and Engineering Department, Gayatri Vidya Parishad College of Engineering for Women, Visakhapatnam, Andhra Pradesh India; 4 Department of Computer Science and Engineering, Vignan’s Institute of Engineering for Women, Visakhapatnam, 530046 India; 5 Department of Electrical and Electronics Engineering, Manipal Institute of Technology, Manipal Academy of Higher Education, Manipal, Manipal, Karnataka 576104 India; 6 School of Computer Applications, KIIT Deemed to be University, Bhubaneswar, India; 7 Department of Electronics and Telecommunications Engineering, Symbiosis Institute of Technology, Pune, India

**Keywords:** Smart cities, Feature selection, Internet of health things, Attack detection, Intrusion detection systems, Harris Hawk optimization, Artificial intelligence, Computer science, Information technology

## Abstract

Internet of Health Things (IoHT) plays a vital role in everyday routine by giving electronic healthcare services and the ability to improve patient care quality. IoHT applications and devices become widely susceptible to cyber-attacks as the tools are smaller and varied. Additionally, it is of dual significance once IoHT contains tools applied in the healthcare field. In the context of smart cities, IoHT enables proactive health management, remote diagnostics, and continuous patient monitoring. Therefore, it is essential to advance a strong cyber-attack detection method in the IoHT environments to mitigate security risks and prevent devices from being vulnerable to cyber-attacks. So, improving an intrusion detection system (IDS) for attack identification and detection using the IoHT method is fundamentally necessary. Deep learning (DL) has recently been applied in attack detection because it can remove and learn deeper features of known attacks and identify unknown attacks by analyzing network traffic for anomalous patterns. This study presents a Securing Attack Detection through Deep Belief Networks and an Advanced Metaheuristic Optimization Algorithm (SADDBN-AMOA) model in smart city-based IoHT networks. The main aim of the SADDBN-AMOA technique is to provide a resilient attack detection method in the IoHT environment of smart cities to mitigate security threats. The data pre-processing phase applies the Z-score normalization method for converting input data into a structured pattern. For the selection of the feature process, the proposed SADDBN-AMOA model designs a slime mould optimization (SMO) model to select the most related features from the data. Followed by the deep belief network (DBN) method is used for the attack classification method. Finally, the improved Harris Hawk optimization (IHHO) approach fine-tunes the hyperparameter values of the DBN method, leading to superior classification performances. The effectiveness of the SADDBN-AMOA method is investigated under the IoT healthcare security dataset. The experimental validation of the SADDBN-AMOA method illustrated a superior accuracy value of 98.71% over existing models.

## Introduction

**Fig. 1:**
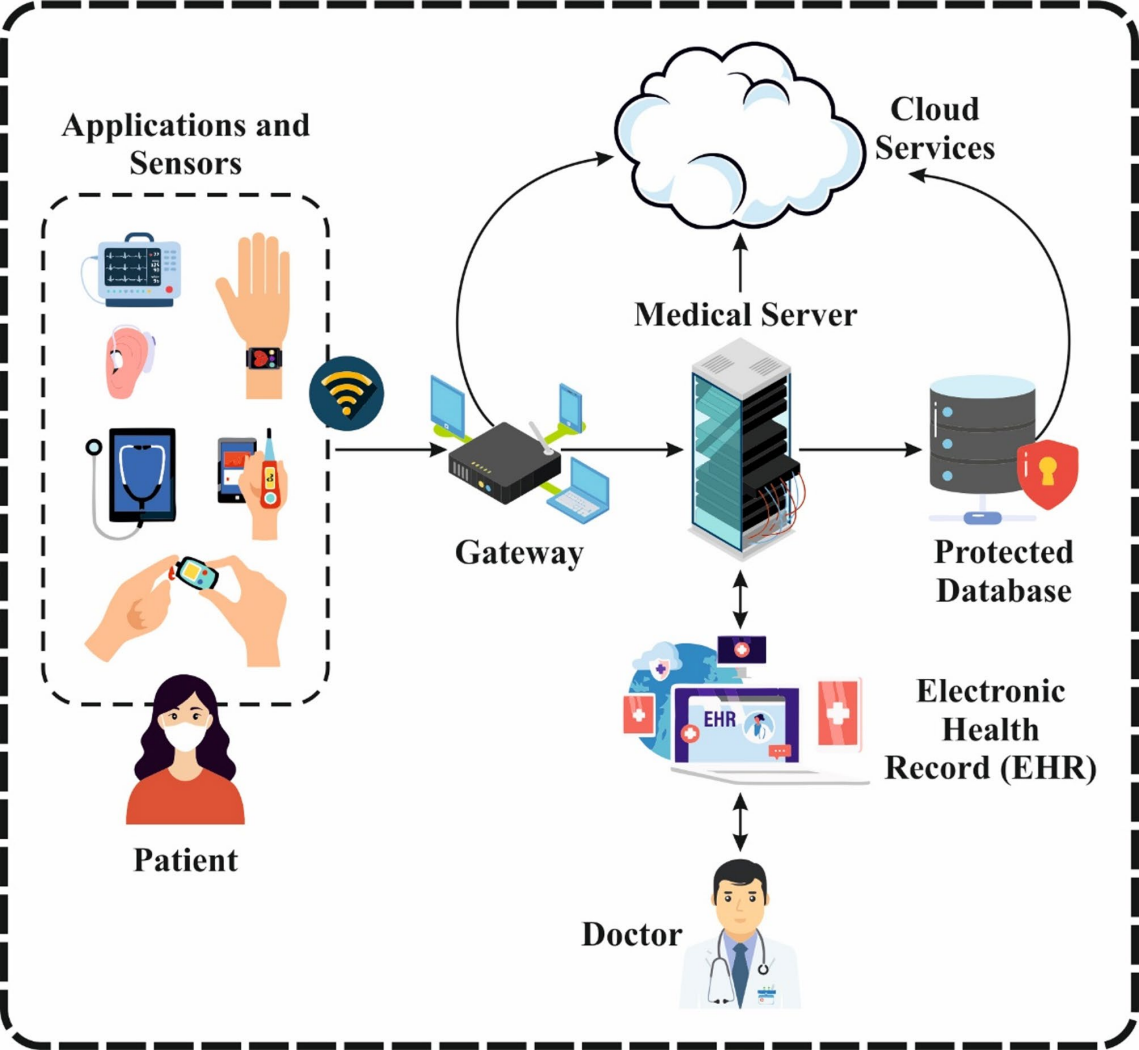
General architecture for the IoHT systems.

The Internet of Things (IoT) is a swiftly developing model. It enables communication between electronic sensors and gadgets linked to the Internet^1^. It is used in numerous smart applications, including industries, homes, transportation, cities, satellites, and healthcare. IoHT is an IoT-aided solution which lets the association between a health care services and patients, namely heart rate, diabetes, electroencephalogram, electrocardiography, and many sensors that encompass breathing (airflow), body temperature, pulse, galvanic skin response, blood pressure, glucometer, electromyography, oxygen in the blood (SPO2) and accelerometer (patient position). IoTs are extensively utilized in various healthcare domains^2^. Many IoT devices employ wireless communication to transmit and receive data, which results in the risk of wireless sensor network (WSN) security breaches. Figure 1 represents the general structure of cyber-attacks in the IoHT systems.

Additionally, the Internet is the primary cause of security risks. It is weak to several types of cyberattacks, namely network sniffing, Denial-of-Service (DoS) attacks, treatment manipulation, and theft of medical records in IoHT landscapes^3^. While several prior studies have effectively established the Internet of Health, intrusion detection is even more problematic due to massive data traffic, accessible network structures, and numerous threat designs^4^. However, several earlier research studies have made such improvements. Applying a powerful procedure will remove the significant, predictable anomalies in the round trip packet times gathered on the internet platform. Due to the real-time communication of a high volume of bio-sensors, there exists the possibility of network intrusion and congestion. The two kinds of network intrusions are intra and inter-network intrusions that often appear in the IoT^5^. Such two kinds of security paradigms may include several synchronized security paradigms such as antiviruses, IDS, and firewalls that allow a system or network to be monitored and send an alarm when malicious activity occurs. IDSs are categorized into three systems: anomaly detection, misuse detection, and hybrid. Intruder detection is one of the crucial developments in ensuring the IoHT network’s security^6^. Investigators revealed many IDS to prevent networks from several attacks. Even if it takes a massive effort by the investigators, IDS still struggles to identify fresh attacks and increase detection precision by decreasing the false detection rate. To solve this problem, numerous investigators are revolving to DL, machine learning (ML), and artificial intelligence (AI) models to detect cyberattacks in IoHT landscapes^7^. These intelligent models present adaptive learning capabilities and deeper feature extraction, making them more effective in detecting complex and previously unseen attack patterns. Furthermore, they enable real-time analysis and decision-making, which is crucial in dynamic IoHT environments.

Smart cities benefit from AI-driven IoHT through efficient hospital resource allocation, pandemic tracking, and personalized medicine. Therefore, ML is inappropriate for recognizing cyberattacks. A better solution for overcoming the limits of ML is to use DL^8^. It can characterize data utilizing the computational approach’s multiple processing layers. Moreover, it will intensely demonstrate primary information and forecast or identify data more precisely than ML due to its multilayer design^9^. However, the straight execution of complicated DL procedures on IoT systems is complex, owing to IoT devices’ inadequate storage, computation, and energy proficiencies. So, deploying DL to detect threats in IoT isn’t a straight way^10^. DL is employed in numerous IDSs for IoHT. DL has an excellent detection level for identifying and transforming assaults. The rapid integration of connected devices in healthcare is revolutionizing patient monitoring and medical services, enabling continuous data collection and real-time health insights. This interconnected environment significantly enhances diagnosis accuracy and personalized care. However, the sensitivity of medical data and the critical nature of healthcare systems make them prime targets for cyber threats. Ensuring robust and effectual protection against these security risks is crucial in maintaining trust and safeguarding patient wellbeing. Advanced techniques are required to address these challenges while assisting the growing complexity of health monitoring technologies.

This study presents a Securing Attack Detection through Deep Belief Networks and an Advanced Metaheuristic Optimization Algorithm (SADDBN-AMOA) model in smart city-based IoHT networks. The main aim of the SADDBN-AMOA technique is to provide a resilient attack detection method in the IoHT environment of smart cities to mitigate security threats. The data pre-processing phase applies the Z-score normalization method for converting input data into a structured pattern. For the selection of the feature process, the proposed SADDBN-AMOA model designs a slime mould optimization (SMO) model to select the most related features from the data. Followed by the deep belief network (DBN) method is used for the attack classification method. Finally, the improved Harris Hawk optimization (IHHO) approach fine-tunes the hyperparameter values of the DBN method, leading to superior classification performances. The effectiveness of the SADDBN-AMOA method is investigated under the IoT healthcare security dataset.

- The SADDBN-AMOA model applies Z-score normalization to standardize input data, improving the dataset’s consistency and stability. This pre-processing step enhances the detection system’s overall performance by mitigating the effects of data variability and scaling differences. As a result, the model attains more reliable and accurate results in detecting security breaches.
- The SADDBN-AMOA method incorporates SMO to perform effectual feature selection, which mitigates the dimensionality of the dataset and removes irrelevant attributes. This optimization improves the detection accuracy by concentrating on the most significant features. Consequently, the approach enhances computational efficiency and the overall performance of the IDS.
- The SADDBN-AMOA approach utilizes the DBN technique to provide a robust classification of security breaches in the IoHT environment, enabling accurate detection of intrinsic attack patterns. This improves the model’s capability of differentiating between normal and malicious activities effectively, significantly improving the reliability and security of healthcare data transmissions.
- The SADDBN-AMOA methodology implements the IHHO method to fine-tune model hyperparameters, improving the detection system’s precision. This optimization mitigates false positives while maximizing overall detection accuracy. Consequently, it strengthens the model’s performance and reliability in detecting security breaches.
- By integrating SMO-based feature selection with DBN classification and IHHO-based hyperparameter tuning, the model achieves a novel synergy that significantly enhances detection accuracy and efficiency. This unique integration addresses the challenges of high-dimensional data and complex IoHT environments. Its novel optimization-driven approach ensures robust, reliable security breach detection, setting it apart from existing methods.

The article’s structure is as follows: Section “Literature review on IoHT attack detection” reviews the literature, Section “Research design and methodology” describes the proposed method, Section “Empirical results and evaluation” presents the evaluation of results, and Section “Conclusion” offers the study’s conclusions.

## Literature review on IoHT attack detection

Kumar et al.^11^ presented an enhanced DL-aided paradigm for detecting cyberattacks and IoMT data authentication to smart healthcare. Firstly, the rooted ensemble learning (EL) model for selecting significant attributes in IoMT mines redundant characteristics and decreases the overfitting possibilities through classifiers. Such measured inputs are served by the presented 1D-convolution long short-term memory (LSTM) (1D-CLSTM) NN for classifying cyber assaults. The random undersampling boosting approach addressed problems such as imbalanced classification. Amjath et al.^12^ suggested a graph-based federated learning (FL) methodology that allows cooperative training among distributed IoHT systems while protecting data privacy. Nevertheless, the growing malware attacks show significant privacy and security difficulties. While centralized GCN and graph attention networks (GAT) are proficient in shaping complex connections to malware recognition, their necessity for centralized data presents scalability and privacy concerns. Benmalek et al.^13^ introduced SNN-IoMT (Stacked Neural Network Ensemble for IoMT Security), an AI-based IDS architecture for securing active IoMT backgrounds. Syeda and Syed^14^ proposed a decentralized, scalable FL method to detect numerous malignant cyber threats in healthcare information. A gated recurrent unit (GRU) DL paradigm was magnificently qualified on ECU-IoHT data through a decentralized client-server-based FL method to detect diverse cyber-attacks. IoHT devices haven’t been efficiently made to protect themselves from internet attacks, which makes them weak to hackers. However, those devices will likely attack patient safety and the healthcare system. Nasayreh et al.^15^ presented a structure to identify cyberattacks and anomalies. The presented combined paradigm uses the K-nearest neighbours (KNN) procedure for classification but utilizes LSTM to extract the features and applies PCA for feature modification and reduction. PCA consequently enriches the significant temporal features detected through the LSTM model. The constraints of the KNN classifier are validated through five-fold cross-validation when creating hyperparameter alterations. Algethami and Alshamrani^16^ projected a hybrid DL-assisted IDS that employs an artificial neural network (ANN) by bidirectional LSTM and GRU frameworks for addressing severe cybersecurity attacks in IoHT. Albattah and Rassam^17^ anticipated a hybrid convolutional LSTM procedure for assuring the IoHT’s dependability in observing applications by identifying abnormalities and confrontational matter in the training data to develop DL techniques.

Also, a countermeasure approach is recommended to safeguard the DL techniques from confrontational threats in the training stage. Consequently, to confirm the consistency of the several applications of IoHT, the testing and training information are essential for that DL method. Bhowmik and Banerjee^18^ intended an edge-enabled effectual privacy-preserving data aggregation (EEPPDA) technique for securing healthcare information. Seized clinical information was encoded with the Paillier homomorphic cryptosystem during this system. Saheed and Chukwuere^19^ presented a privacy-preserving and interpretable intrusion detection model for CPS-IIoT using Pearson correlation, agglomerative clustering, and BiLSTM with scaled dot-product attention for improved accuracy and security. Gupta et al.^20^ proposed a tree classifier-based intrusion detection model for IoMT networks that ensures fast and accurate anomaly detection while preserving patient privacy. Saheed and Misra^21^ developed an explainable and privacy-preserving deep neural network (DNN) methodology by utilizing SHapley Additive exPlanations (SHAP) for accurate and interpretable anomaly detection in CPS-IoT networks. Gupta et al.^22^ developed a lightweight blockchain-based IDS for IoMT networks that ensures data integrity, privacy, and effective detection of diverse cyberattacks with minimal computational overhead. Khan et al.^23^ presented a lightweight hybrid federated SVM and trust management model that ensures secure, privacy-preserving, and efficient anomaly detection in smart healthcare systems. Saheed, Abdulganiyu, and Ait Tchakoucht^24^ developed a hybrid ensemble learning model using principal component analysis (PCA) and grey wolf optimizer (GWO) for accurate and real-time intrusion detection in supervisory control and data acquisition (SCADA) systems with industrial sensor networks (ISNs). Darabkh and Al-Akhras^25^ proposed an energy-efficient clustering and routing protocol using the marine predators’ algorithm (MPA) method to extend the lifespan and improve the performance of IoT sensor networks in smart city applications. Saheed et al.^26^ proposed a lightweight intrusion detection model using transfer learning (TL) with ResNet50-CNN1D and Adagrad optimizer for accurate and efficient cyber-attack detection in CPS. Dandamudi, Sajja, and Khanna^27^ explored how AI improves cybersecurity and network efficiency in the U.S., improving threat detection, response times, and bandwidth management. Saheed and Chukwuere^28^ presented an explainable, efficient TL model for detecting zero-day botnet attacks in the Internet of Vehicles (IoV), improving accuracy and transparency while reducing reliance on large labelled datasets. Manivannan and Senthilkumar^29^ developed an adaptive recurrent neural network (RNN) model with a fox optimizer for accurate and efficient network intrusion detection. Saheed, Misra, and Chockalingam^30^ developed a low-cost, unsupervised IDS using autoencoder-based feature reduction with DCNN and LSTM for accurate anomaly detection in industrial control systems (ICS) without prior network knowledge.

Despite various advances in IDS for IoMT, IoHT, CPS, and ICS, several research gaps still exist. Many models depend heavily on labelled data, which is often scarce or imbalanced, limiting the detection of novel attacks. Privacy concerns persist, specifically in federated and centralized learning approaches, affecting scalability and data sharing. Also, various DL techniques encounter threats with interpretability and computational overhead, restricting deployment on resource-constrained devices. Feature selection and extraction methods sometimes lack robustness, affecting overall accuracy. Moreover, many proposed results do not fully address real-time detection requirements or adaptability to growing threats, highlighting a clear research gap while addressing it.

## Research design and methodology

**Fig. 2:**
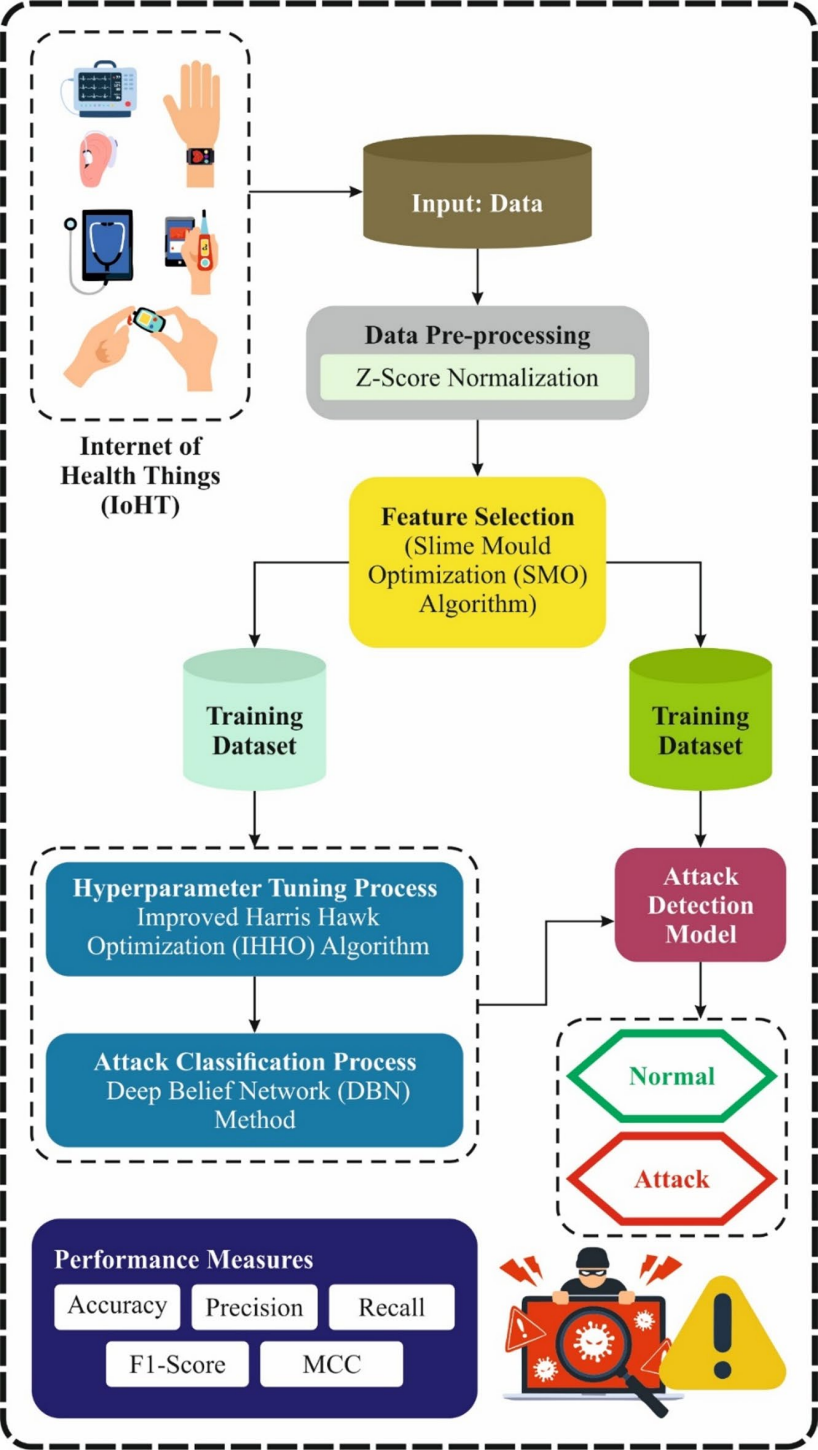
Overall process of SADDBN-AMOA model.

This paper proposes a novel SADDBN-AMOA technique for smart city-based IoHT networks. The main aim of the SADDBN-AMOA technique is to provide a resilient attack detection method in the IoHT environment of smart cities to mitigate security threats. It contains data pre-processing, feature optimizer, DL with attack detection, and model tuning with IHHO. Figure 2 depicts the entire process of the SADDBN-AMOA model.

### Data Pre-processing through normalization

The data pre-processing step initially applies the Z-score normalization method for transforming input data into a structured pattern^31^. This method is chosen for its efficiency in standardizing data by converting features to have a mean of zero and a standard deviation of one, which assists in stabilizing the training process and speeds up convergence. Unlike min-max scaling, which can be sensitive to outliers, Z-score normalization is more robust as it accounts for data distribution, mitigating the impact of extreme values. This method ensures that features with diverse units or scales contribute equally to the model, averting bias towards variables with larger ranges. Additionally, it enhances the performance of models such as DBN, which usually assume distributed input data. Hence, this improves the model’s stability, accuracy, and generalization compared to other scaling techniques.

Z-score normalization, or standard score normalization, is a data pre-processing model that converts feature values by centring them near the mean with a standard deviation of 1. Concerning attack detection in IoHT atmospheres, this model assistances guarantees that each feature corresponds to the detection method, particularly after they have dissimilar units or scales. This is important for ML methods aware of feature sizes, like neural networks, SVM, or k-NN. This model improves model accuracy, performance, and convergence speed by decreasing bias from leading features. In IoHT methods, while sensor data might differ extensively, this standardization enhances the dependability of intrusion detection or anomaly. It also facilitates more efficient classification and feature selection in resource-restricted IoHT devices.

### Feature optimization with SMO model

For selecting the feature process, the proposed SADDBN-AMOA model utilizes the SMO method to choose the most related features from the data^32^. This model was selected for its robust global search capability and efficient exploration-exploitation balance, which assists in avoiding premature convergence, which is common in other algorithms. Unlike conventional methods like genetic algorithms (GA) or particle swarm optimization (PSO), SMO illustrates faster convergence and improved adaptability to intrinsic, high-dimensional data. Its bio-inspired mechanisms effectively detect the most relevant features, mitigating dimensionality without losing critical data. This results in an enhanced accuracy of the model and reduced computational cost. Moreover, the simplicity and fewer parameters of the model make it easier to implement and tune, presenting a robust and reliable approach for optimizing feature subsets in IoHT security applications. SMO is chosen due to its latest success as a meta-heuristic model stimulated by foraging behaviour and slime mould dispersion. The SMO has three stages: wrap, approach, and search for food. The mathematical representation of all expressions is mentioned below.

Stage 1-Approaching Food: Subject to the smell (scent) that food creates, SM might approach it. Like the technique of using mathematical equations to observe the mode of contraction approaching behaviours, the succeeding equations are presented to mimic it. The equation that inspires the SM to approach food is calculated by Eq. (1)

1 $$\:\overrightarrow{X}\left(t+1\right)=\left\{\begin{array}{ll}{\overrightarrow{X}}_{b}\left(t\right)+\overrightarrow{{v}_{b}}\times\:\left(\overrightarrow{W}\times\:{\overrightarrow{X}}_{A}\left(t\right)-{\overrightarrow{X}}_{B}\left(t\right)\right),&\:r<p\\\:\overrightarrow{{v}_{c}}\cdot\:\overrightarrow{X}\left(t\right),&\:r\ge\:p\end{array}\right.$$

Here, $\:\overrightarrow{{v}_{b}}$ refers to parameters ranging from $\:-1$ to $\:+1$, and $\:\overrightarrow{{v}_{c}}$ linearly reduces from 1 to $\:0$ for the last specific iteration. $\:W$ denotes the slime mould’s weight, and $\:t$ represents the iteration that is now in progress. From the SM, the $\:{\overrightarrow{X}}_{A}\left(t\right)$ and $\:{\overrightarrow{X}}_{B}\left(t\right)$ are selected randomly. The $\:\overrightarrow{X}$ characterizes the location of the SM, while $\:{\overrightarrow{X}}_{b}$ specifies the present individual’s location at the odour focus of the meal is greater. Equation (2) is utilized for calculating the $\:p$-value.

2 $$\:p=\text{t}\text{a}\text{n}\text{h}\left|S\left(i\right)-DF\right|\:$$

The fitness of $\:\overrightarrow{X}$ was characterized by $\:S\left(i\right)$, whereas the maximal fitness gained through each iteration $\:S(i\in\:\text{1,2},3\dots\:\dots\:\dots\:n)$ is signified by DF. The $\:\overrightarrow{{v}_{b}}$ value varies from − a to $\:a$, and the $\:a$ value is calculated using Eqs. (3) and (4).

3 $$\:\overrightarrow{{v}_{b}}\in\:\left[-a\:to\:a\right]$$

4 $$\:a=arc\text{t}\text{a}\text{n}\text{h}\left(-\left(\frac{t}{{\text{m}\text{a}\text{x}}_{iter}}\right)+1\right)$$

The weight ($\:W$) value is measured by Eq. (5) as shown,

5 $$\vec{W}~\left( {SmellIndex\left( i \right)} \right) = \left\{ {\begin{array}{*{20}l} {1 + r \cdot log\left( {\frac{{b_{F} - S\left( i \right)}}{{b_{F} - w_{F} }} + 1} \right),} \hfill & {if\,S\left( i \right) < Med\left[ S \right]} \hfill \\ {1 - r \cdot log\left( {\frac{{b_{F} - S\left( i \right)}}{{b_{F} - w_{F} }} + 1} \right),} \hfill & {otherwise} \hfill \\ \end{array} } \right.$$

6 $$\:SmellIndex\left(SI\right)=Sort\left(S\right)$$

The above equation has the following elements: $\:r$ embodies randomly generated values within the range $\:\left[\text{0,1}\right]$, which specifies that $\:S\left(i\right)$ is acknowledged for ranking in the higher portion. The $\:{b}_{f\:}$and $\:{w}_{f}$ characterize the best and worst values of fitness gained within the present iterative method. The $\:{\text{m}\text{a}\text{x}}_{iter}$ specifies the maximal iteration amount. $\:Med\left[S\right]$refers to median operator representation. The $\:SmellIndex\left(SI\right)$displays the values of fitness in sequential order.

Stage 2-Food Wrapping: The quality and nature of the SM search model are impacted. A position’s weight will rise after a considerable focus on food is greater. It is compelled to search another area when the attention is lower as the area’s weight falls. This stage consists of upgrading the position of the SM utilizing Eq. (7):

7 $$\:\overrightarrow{X}\left(t+1\right)=\left\{\begin{array}{ll}rand\cdot\:\left(UB-LB\right)+LB,&\:rand<z\\\:{\overrightarrow{X}}_{b}\left(t\right)+\overrightarrow{{v}_{b}}\times\:\left(\overrightarrow{W}\times\:{\overrightarrow{X}}_{A}\left(t\right)-{\overrightarrow{X}}_{B}\left(t\right)\right),&\:r<p\\\:\overrightarrow{{v}_{c}}\cdot\:\overrightarrow{X}\left(t\right),&\:r\ge\:p\end{array}\right.\:$$

Whereas $\:z$ denotes probability applied to attack a balance between exploitation and exploration, and $\:UB$ and $\:LB$ represent upper and lower bound limits.

Stage 3-Food Grabbling: SM moves to places with high food attention. $\:W,$ $\:\overrightarrow{{v}_{b}},$ and $\:\overrightarrow{{v}_{c}}$ characterize changing venous widths. $\:\overrightarrow{{v}_{b}}$ and $\:\overrightarrow{{v}_{b}}$ swing correspondingly between $[-a, a]$ and [-1, 1]. For example, $\:\overrightarrow{{v}_{b}}$ and $\:\overrightarrow{{v}_{b}}$ become nearer to 0 when iteration improves.

The fitness function (FF) deliberates the classification accuracy and the preferred features. It maximizes the classification accuracy and reduces the set size of the selected attributes. Then, the succeeding FF is deployed to approximate unique results, as presented in Eq. (8).

8 $$\:Fitness=\alpha\:\cdot\:\:ErrorRate+\left(1-\alpha\:\right)\cdot\:\frac{\#SF}{\#All\_F}$$

Now, $\:ErrorRate$ symbolizes the classification $\:ErrorRate$ utilizing the chosen features. $\:ErrorRate$ is calculated as the incorrect percentage categorized to the sum of classifications produced, identified as the value among (0,1), $\:\#SF$ denotes preferred feature counts, and $\:\#All\_F$ stands for the complete quantity of features in the novel data set. $\:\alpha\:$ is used for controlling the prominence.

### DL for attack detection

**Fig. 3:**
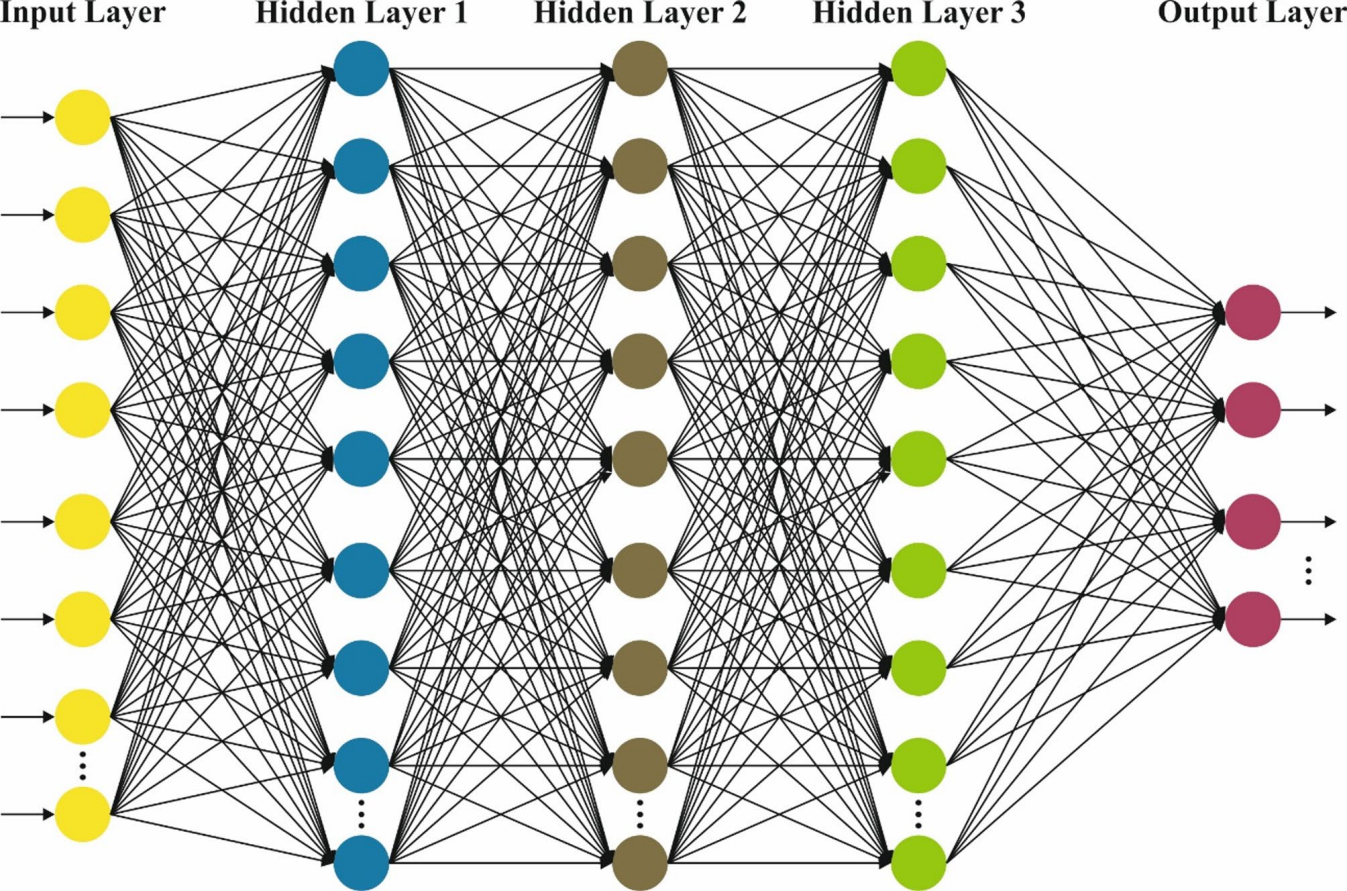
DBN architecture.

The DBN method is followed for the attack classification process^33^. This method is chosen for its effectual capability in learning hierarchical feature representations from complex and high-dimensional data, which is common in IoHT environments. Unlike conventional classifiers, DBN efficiently extracts deep features without extensive manual pre-processing, thus enhancing detection accuracy. The model also integrates unsupervised pretraining with fine-tuning, improving generalization and mitigating overfitting. Compared to models such as standard neural networks or SVMs, DBNs are more efficient in capturing complex patterns and dependencies in security breach data. This results in robust and reliable classification performance, making DBNs appropriate for the dynamic and diverse nature of IoHT security threats. Figure 3 demonstrates the structure of DBN.

DBNs are a DL method designed to capture composite hierarchic data representation. They include numerous stochastic, latent variables layers and can learn complex designs from higher-dimensional data. DBNs are valuable in settings, whereas feature learning and reducing the dimensions are essential, like network intrusion detection. This part examines the numerical basics, architecture, and DBNs application, highlighting their part in recognizing networking assaults. A DBN comprises numerous Restricted Boltzmann Machine (RBMs) layers and the supervised method’s last layer, often softmax classifiers. The DBN structure consists of the following elements:

RBMs: All RBMs in the DBN are two-layered, stochastic NN comprising a visible layer (VL) and a hidden layer (HL). The VL characterizes the input data, whereas the HL takes high-level characteristics. The network is trained to maximize the data probability, and the links among the layers are aimless. The main modules are:

- VL: Characterizes the detected information and has real-valued or binary elements.
- HL: Signifies the feature learning from the input information and takes concealed designs.
- Weights: The relations between the HL and VL are characterized by weighting that is learned in training.

The RBM’s energy function is provided by:

9 $$\:E\left(\nu\:,\:h\right)=-{\sum\:}_{i}{b}_{i}{\nu\:}_{i}-{\sum\:}_{j}{c}_{j}{h}_{j}-{\sum\:}_{i,j}{w}_{ij}{\nu\:}_{i}{h}_{j}\:$$

Whereas $\:\nu\:$ and $\:h$ represent hidden and visible elements, $\:{w}_{ij}$ are weighted, and$\:\:{b}_{i}$ and $\:{c}_{j}$ are biased.

*Stacking RBMs*: They are produced by stacking numerous RBMs in the deeper structure. All RBMs are trained layer-by-layer, whereas the output only RBM acts as the input to the following.

Last Layer of Classification: Afterward, RBM’s pretraining, the last supervised layer, is added to carry out regression or classification assignments. The learning procedure in DBNs consists of dual primary stages: fine-tuning and pretraining.

Pretraining: This stage consists of training all RBMs autonomously utilizing unsupervised learning. The aim is to learn the weights, which maximizes the data probability. The model of contrastive divergence is usually applied to train RBMs:

10 $$\:\varDelta\:{w}_{ij}=\eta\:\left(\right\{{v}_{i}{h}_{j}{\}}_{data}-\left\{{v}_{i}{h}_{j}{\}}_{model}\right)\:$$

Here, $\left\langle {\nu _{i} h_{j} } \right\rangle _{{data}}$ refers to the predictable value of the output from the information’s hidden and visible components, and $\left\langle {\nu _{i} h_{j} } \right\rangle _{{model}}$ denotes the predicted value from the model distribution.

Finetuning: Afterward, the complete DBN is finetuned. This includes upgrading the weighting of the last layer of classification and, possibly, the weighting of the RBMs. This stage utilizes gradient descent to reduce the function of loss:

11 $$\:L=-{\sum\:}_{i}({y}_{i}\:\text{l}\text{o}\text{g}\left({\widehat{y}}_{i}\right)+\left(1-{y}_{i}\right)\text{l}\text{o}\text{g}\left(1-{\widehat{y}}_{i}\right)\:$$

Meanwhile, $\:{y}_{i}$ denotes true labels, and $\:{\widehat{y}}_{i}$ represents forecast likelihoods.

DBNs may be used for learning the hierarchic representation of networking traffic data, allowing the recognition of composite attacking designs. The capability to remove and learn from higher-level attributes makes DBNs suitable for identifying unknown or known intrusions. By using DBNs, networking intrusion detection methods can take advantage of enhanced dimensionality reduction and feature extraction, improving their capability to recognize subtle patterns and anomalies suggestive of mischievous action. DBNs’ deeper hierarchical architecture permits further complex classification and understanding of networking traffic, possibly resulting in high precision and strength in identifying network attacks. Generally, DBNs provide a robust architecture to learn hierarchic feature representation. Their capability for modelling composite designs and decreasing dimensions makes them a helpful device in the current strength to improve cybersecurity through progressive DL methods.

### Model tuning with IHHO approach

Eventually, the IHHO technique adjusts the DBN model’s hyperparameter values, resulting in greater classification performance^34^. This model is chosen for its superior exploration and exploitation balance, which effectively avoids local optima and ensures global search efficiency. This model enables dynamic adaptation and faster convergence compared to conventional optimization methods, namely GA or PSO, replicating the cooperative hunting strategy of Harris hawks. Its improved ability to fine-tune hyperparameters results in enhanced detection accuracy and mitigated false positives in complex IoHT environments. Moreover, the flexibility and robustness of the model make it appropriate for optimizing DL models, ensuring reliable performance even with high-dimensional and noisy data.

The HHO model is one of the population-based optimizer models that emulate the cooperative behaviour and hunting approach of the predacious bird HHO in the foraging process. Amongst the running of the model, the HHO has the candidate solution, and the target approach is the optimum solution with iterations. The HHO model might be segmented into 3 phases: local exploitation, conversion among exploitation and exploration, and global exploration.

The intention of the HHO model generates an issue with the following simplicity:

- The tactic of arbitrarily initializing the population is employed. The arbitrary creation of the population results in the arbitrary distribution of created HHO individuals in the searching area, which results in the technique’s uncertainty.
- In the method’s iterative procedure, only the data of the finest location is employed, which affects the complete model, falling into a local optimal when the existing finest location has a local optimal.
- During the search process, the HHO model chooses the optimum acquiring approach completely reliant on the parameters $\:\lambda\:$ and $\:E.$ These dual parameters will instantly impact the HHO’s ending outcome. The decrease approach of escaping energy $\:E$ should attempt to fit the modification of the target’s energy in the procedure of escaping the actual situation, and the parameter value $\:\lambda\:$ additionally guarantees the unpredictability. Otherwise, it will decrease the model’s capability to exploit and search.

### Logistic-tent chaotic mapping initializing populations

This model is accepted to resolve the irregular distribution of population initialization. The examination has depicted that integrating several lower-dimension chaotic mapping techniques to establish a complex, chaotic technique might effectively resolve the issues.

12 $$\:{X}_{n+1}=\mu\:{X}_{n}\left(1-{X}_{n}\right),\:n=\text{1,2}\cdots\:$$

Here, $\:\mu\:$ represents the control parameter, and $\:X$ depicts the system variable. Logistic chaotic mapping has similar issues once the system parameters range from zero to one.

13 $$\:{X}_{n+1}=\left\{\begin{array}{l}\frac{{X}_{n}}{\alpha\:},{X}_{n}\in\:\left[0,\:\alpha\:\right)\\\:\frac{(1-{X}_{n})}{(1-\alpha\:)},{X}_{n}\in\:\left[\alpha\:,\:1\right]\end{array}\right.\:$$

Here, X specifies the system variable and $\:\alpha\:\in\:\left(\text{0,1}\right)$. It concerns some controlling parameters and restricted intervals of chaos.

According to the standard of Logistic-tent chaotic maps, the logistic and tent combined chaotic maps method might be formed by taking the outputs of Logistic maps as the input of Tent maps and iteratively analyzing them. This complex chaotic maps method shows the speed of iteration, and auto-correlation effectively resolves the arbitrary initialization of the population.

14 $$\:{X}_{n+1}=\left\{\begin{array}{l}mod\left[\mu\:{X}_{n}\left(1-{X}_{n}\right)+\frac{\left(4-\mu\:\right){X}_{n}}{2}\right],\:{X}_{n}<0.5\\\:mod{\:\left[\beta\:r{X}_{n}\left(1-{X}_{n}\right)+\frac{\left(4-\mu\:\right)\left(1-{X}_{n}\right)}{2}\right],\:X}_{n}\ge\:0.5\end{array}\right.\:$$

$\:n$ represents the location of arbitrarily generated HHO, and $\:{X}_{n+1}$ indicates the location of $\:n$th HHO once chaotic mapping is composited.

### Population hierarchies improve search strategy

This approach has a type of population hierarchy, choosing the first five optimum locations in every iteration process rather than the one optimum location accepted initially. This increases the interaction among populations and effectively decreases the risky model falling into the local optimal.

15 $$\:{X}_{rabbit}=\frac{\sum\:_{i=a}^{e}f\left({X}_{ir}\left(t\right)\right)}{\sum\:_{j=a}^{e}f\left({X}_{jr}\left(t\right)\right)}\cdot\:X\left(t\right)\:$$

$\:a,b,$ $\:c,d$, and $\:e$ are the top 3 optimum locations regarding fitness value, $\:{X}_{rabbit}$ specifies the optimum location chosen in every iteration, $\:t$ represents iteration counts, and $\:f\left({X}_{ir}\left(t\right)\right)$ indicates the fitness value of the optimum location in the 10-th iteration.

### Enhancement of reducing escape energy approach

It is motivated by the fractional order predator-prey dynamical technique with housings; an advanced model is projected for escaping the energy-reducing approach, which presents a contraction function in the energy-reducing equations to prevent the model from getting stuck in local optima by ensuring the escaping energy $\:E$ remains lower in mid and late iterations.

16 $$\:{E}_{1}=2\cdot\:rand\cdot\:{e}^{-\left(\frac{\pi\:}{2}\cdot\:\left(\frac{c}{T}\right)\right)}\:$$

17 $$\:E=2{E}_{0}\cdot\:{E}_{1}$$

The IHHO model initiates an FF to obtain heightened classification performance. It summarizes progressive numbers to characterize the enriched outcome of the candidate solutions. In this paper, the minimization of the classification rate of error is imitated as the FF, as provided in Eq. (18).

18 $$\begin{aligned} fitness\left( {x_{i} } \right) & = ClassifierErrorRate\left( {x_{i} } \right) \\ & = \frac{{no\:of\:misclassified\:samples}}{{Total\:no\:of\:samples}} \times 100 \\ \end{aligned}$$

## Empirical results and evaluation

The experimental validation of the SADDBN-AMOA method is examined under the IoT healthcare security dataset^35^. This dataset comprises 188,694 instances under normal and attack classes, as depicted in Table 1. It contains 50 features in total, but only 35 features are selected.

**Table 1:** Details of the dataset.

Classes	Number of Instances
Normal (Patient Monitoring, Environment Monitoring)	108,568
Attack	80,126
Total Number of Instances	188,694

**Fig. 4:**
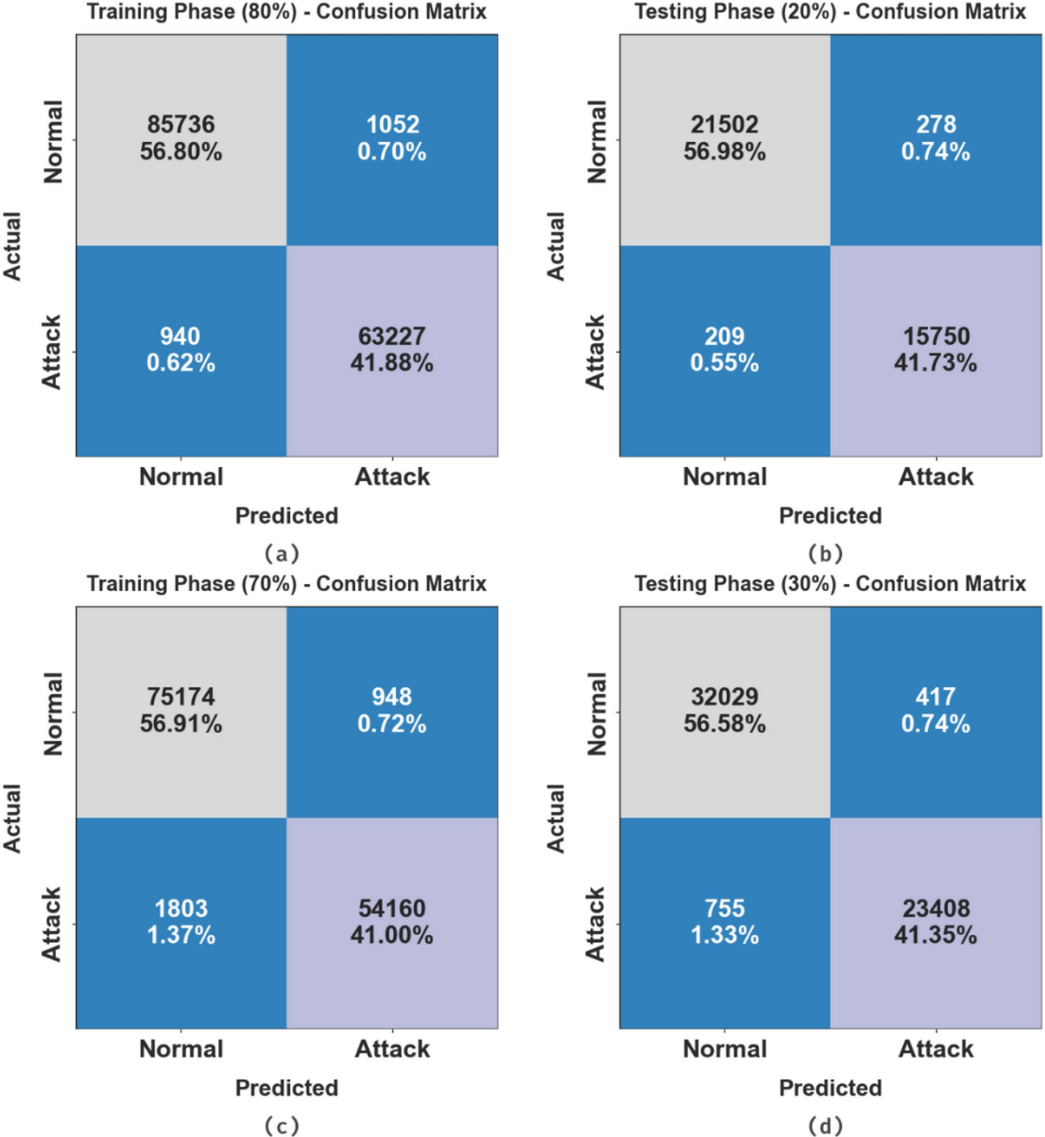
Confusion matrix of SADDBN-AMOA model under TRPHE and TSPHE of (**a**-**b**) 80:20 (**c**-**d**) 70:30.

Figure 4 depicts the confusion matrix of the SADDBN-AMOA approach under 80:20 and 70:30 of TRPHE/TSPHE. The results show that the SADDBN-AMOA method is proficient in recognizing and identifying all two classes.

**Fig. 5:**
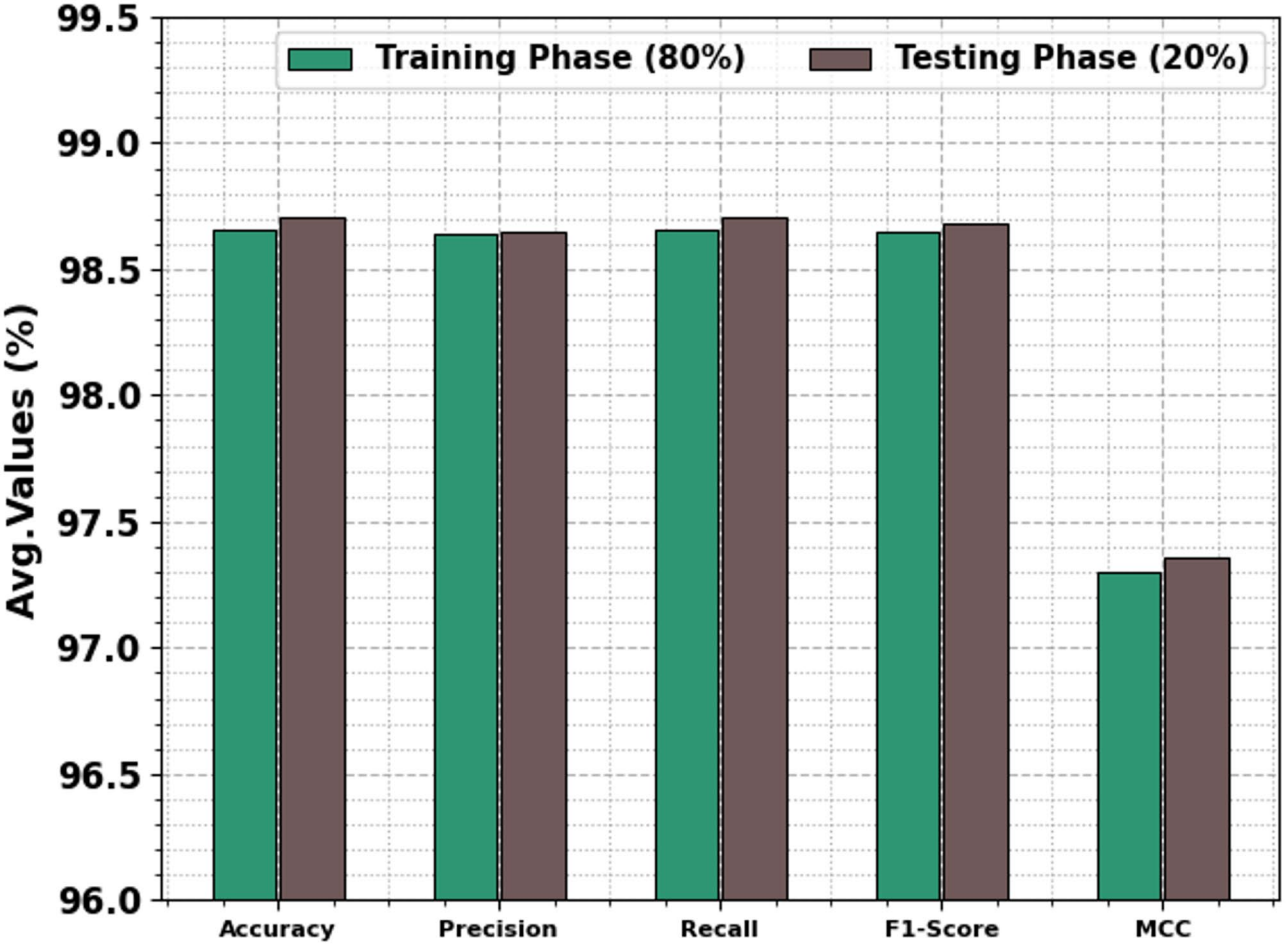
Average of SADDBN-AMOA method on 80%TRPHE and 20%TSPHE.

Table 2; Fig. 5 represent the attack detection of the SADDBN-AMOA approach under 80:20 of TRPHE/TSPHE. The outcome stated that the SADDBN-AMOA approach has appropriately classified all the dissimilar classes. Based on 80% TRPHE, the proposed SADDBN-AMOA approach gains average $\:acc{u}_{y}$, $\:pre{c}_{n}$, $\:rec{a}_{l}$, $\:{F1}_{score},\:$and $\:MCC$ of 98.66%, 98.64%, 98.66%, 98.65%, and 97.30%, respectively. Followed by, depend upon 20%TSPHE, the proposed SADDBN-AMOA technique realizes average $\:acc{u}_{y}$, $\:pre{c}_{n}$, $\:rec{a}_{l}$, $\:{F1}_{score},\:$and $\:MCC$ of 98.71%, 98.65%, 98.71%, 98.68%, and 97.36%, respectively.

**Table 2:** Attack detection of SADDBN-AMOA method under 80:20 of TRPHE/TSPHE.

Class Labels	Accu_y_	Prec_n_	Reca_l_	F1_score_	MCC
TRPHE (80%)					
Normal	98.79	98.92	98.79	98.85	97.30
Attack	98.54	98.36	98.54	98.45	97.30
Average	**98.66**	**98.64**	**98.66**	**98.65**	**97.30**
TSPHE (20%)					
Normal	98.72	99.04	98.72	98.88	97.36
Attack	98.69	98.27	98.69	98.48	97.36
Average	**98.71**	**98.65**	**98.71**	**98.68**	**97.36**

**Fig. 6:**
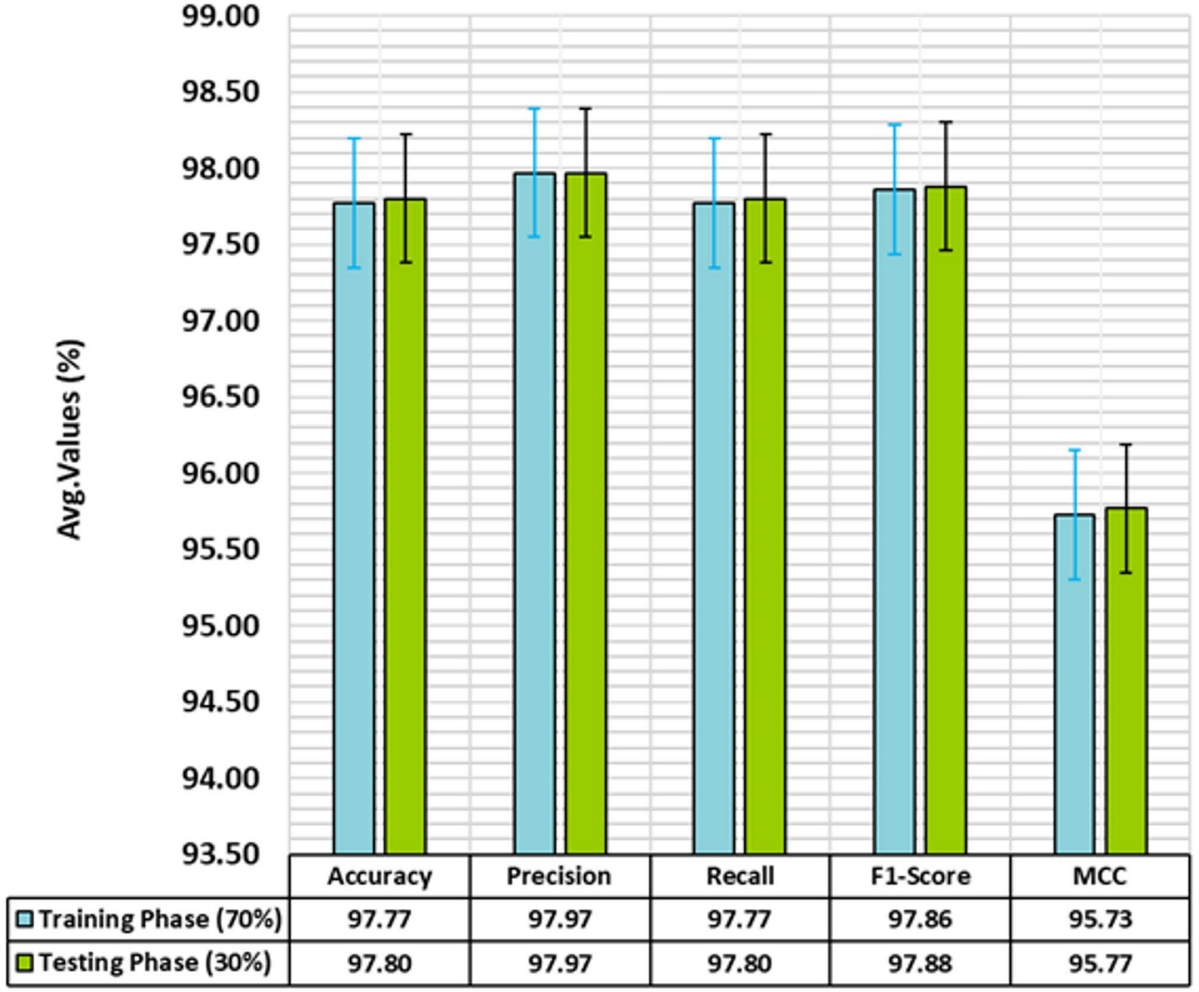
Average of SADDBN-AMOA model on 70%TRPHE and 30%TSPHE.

Table 3; Fig. 6 present the attack detection of the SADDBN-AMOA approach under 70:30 of TRPHE/TSPHE. Based on 70% TRPHE, the proposed SADDBN-AMOA approach attains average $\:acc{u}_{y}$, $\:pre{c}_{n}$, $\:rec{a}_{l}$, $\:{F1}_{score},\:$and $\:MCC$ of 97.77%, 97.97%, 97.77%, 97.86%, and 95.73%, correspondingly. Moreover, on 30%TSPHE, the proposed SADDBN-AMOA method attains average $\:acc{u}_{y}$, $\:pre{c}_{n}$, $\:rec{a}_{l}$, $\:{F1}_{score},\:$and $\:MCC$ of 97.80%, 97.97%, 97.80%, 97.88%, and 95.77%, respectively.

**Table 3:** Attack detection of SADDBN-AMOA model under 80:20 of TRPHE/TSPHE.

Class Labels	Accu_y_	Prec_n_	Reca_l_	F1_score_	MCC
TRPHE (70%)					
Normal	98.75	97.66	98.75	98.20	95.73
Attack	96.78	98.28	96.78	97.52	95.73
Average	**97.77**	**97.97**	**97.77**	**97.86**	**95.73**
TSPHE (30%)					
Normal	98.71	97.70	98.71	98.20	95.77
Attack	96.88	98.25	96.88	97.56	95.77
Average	**97.80**	**97.97**	**97.80**	**97.88**	**95.77**

**Fig. 7:**
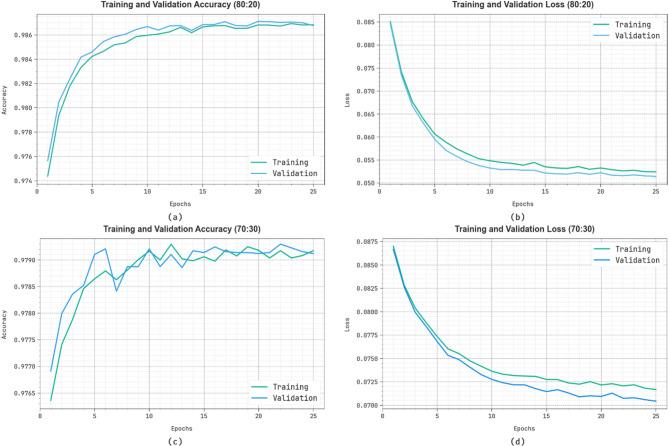
(**a**-**c**) $\:Acc{u}_{y}$ analysis on 80:20 and 70:30 and (**b**-**d**) Loss graph on 80:20 and 70:30

Figure 7 demonstrates the classifier results of the SADDBN-AMOA approach at 80:20 and 70:30. Figure 7a and c proves the $\:acc{u}_{y\:}$values of the SADDBN-AMOA approach. The figure informs that the proposed methodology gains growing values above raising epochs. The rising validation across training exhibitions that the SADDBN-AMOA approach learns capably on the test dataset. Finally, Fig. 7b and d clarifies the loss investigation of the SADDBN-AMOA approach. The results show that the SADDBN-AMOA model accomplishes a closer validation and training loss analysis. It is experimental that the proposed model learns proficiently on the test dataset.

**Fig. 8:**
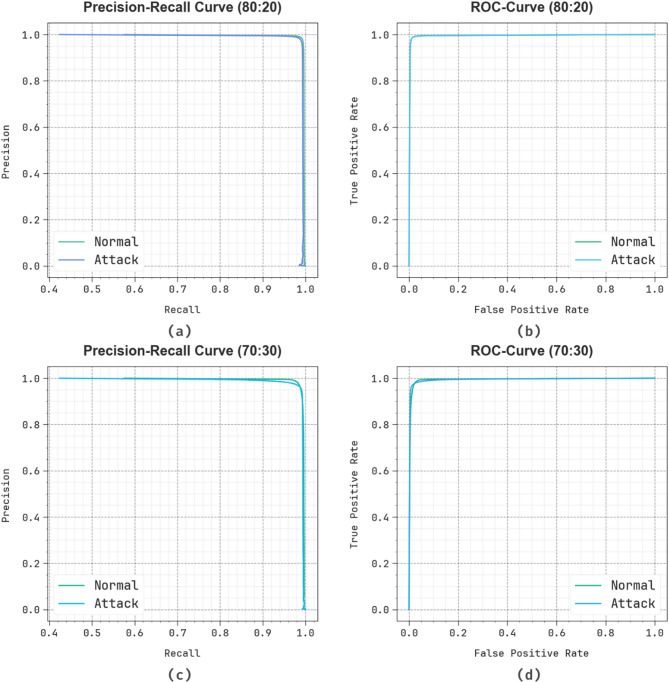
(**a**-**c**) PR graph on 80:20 and 70:30 and (**b**-**d**) ROC graph on 80:20 and 70:30.

Figure 8 proves the classifier results of the SADDBN-AMOA methodology on 80:20 and 70:30. Figure 8a and c establishes the PR values of the SADDBN-AMOA method. The results stated that the proposed methodology results in growing PR values. Simultaneously, it is evident that the SADDBN-AMOA method can achieve better PR analysis in all classes. Ultimately, Fig. 8b and d explains the ROC inspection of the SADDBN-AMOA method. The figure described that the SADDBN-AMOA method was examined for greater ROC analysis. Moreover, the proposed method can extend the maximum ROC analysis on all class labels.

**Fig. 9:**
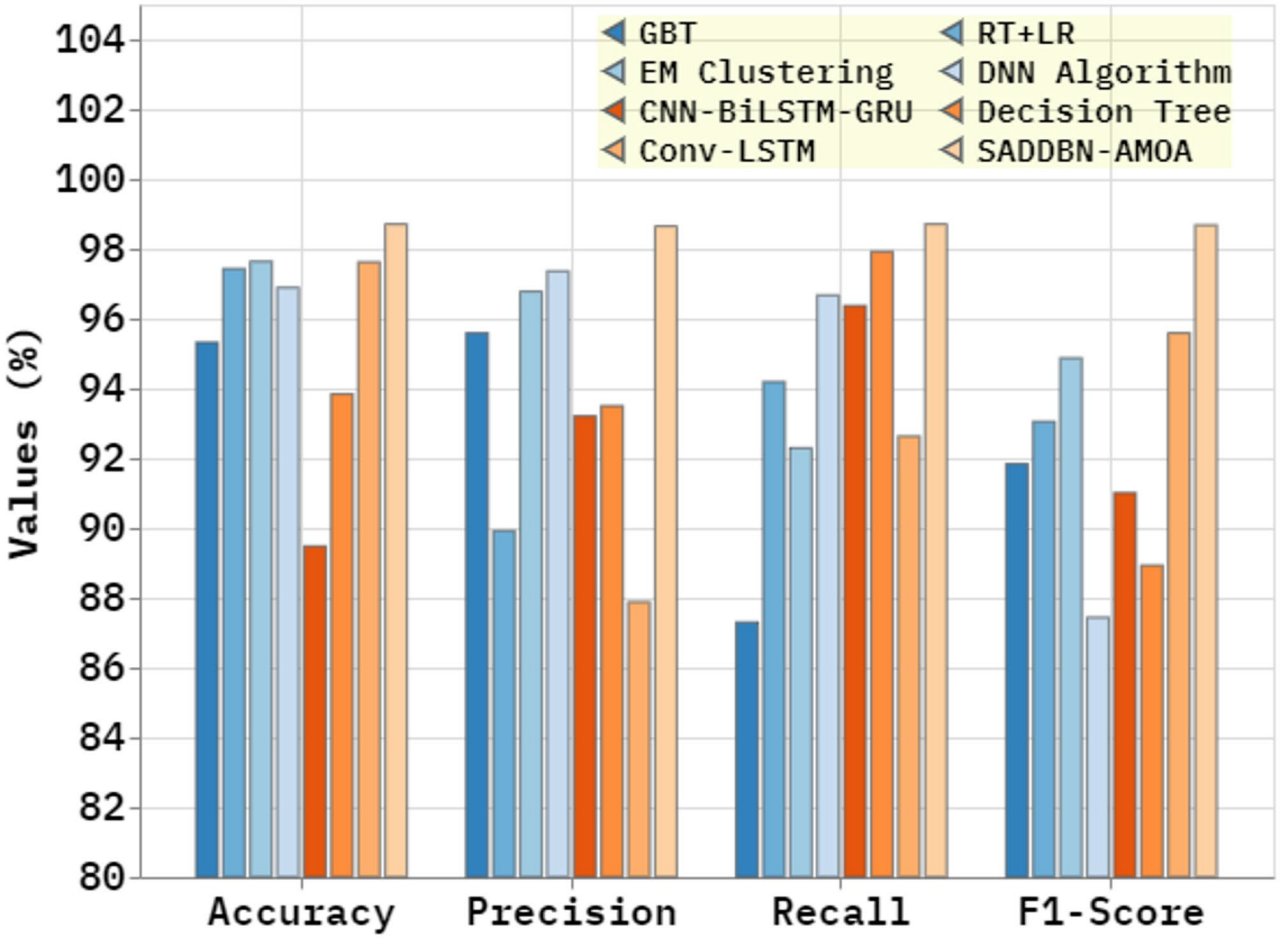
Comparative study of SADDBN-AMOA method.

The comparison study of the SADDBN-AMOA approach with existing models is illustrated in Table 4; Fig. 9^36,37^. The simulation result specified that the SADDBN-AMOA approach outperformed optimal performances. Based on $\:acc{u}_{y}$, the SADDBN-AMOA approach has greater $\:acc{u}_{y}$ of 98.71% where the GBT, RT + LR, EM Clustering, DNN, CNN-BiLSTM-GRU, Decision Tree, and Conv-LSTM approaches obtained lesser $\:acc{u}_{y}$ of 95.32%, 97.43%, 97.64%, 96.89%, 89.48%, 93.84%, and 97.62%, respectively. Besides, depending upon $\:Pre{c}_{n}$, the SADDBN-AMOA technique has maximum $\:Pre{c}_{n}$ of 98.65% where the GBT, RT + LR, EM Clustering, DNN, CNN-BiLSTM-GRU, Decision Tree, and Conv-LSTM approaches gained minimal $\:Pre{c}_{n}$ of 95.59%, 89.92%, 96.78%, 97.36%, 93.21%, 93.50%, and 87.88%, correspondingly. Moreover, based on $\:Rec{a}_{l}$, the SADDBN-AMOA methodology has a higher $\:Rec{a}_{l}$ of 98.71%. In contrast, the GBT, RT + LR, EM Clustering, DNN, CNN-BiLSTM-GRU, Decision Tree, and Conv-LSTM approaches accomplished lower $\:Rec{a}_{l}$ of 87.30%, 94.19%, 92.29%, 96.67%, 96.37%, 97.92%, and 92.63%, respectively. Finally, based on $\:{F1}_{score}$, the SADDBN-AMOA methodology has a higher $\:{F1}_{score}$ of 98.68%. At the same time, the GBT, RT + LR, EM Clustering, DNN, CNN-BiLSTM-GRU, Decision Tree, and Conv-LSTM approaches achieved minimum $\:{F1}_{score}$ of 91.84%, 93.05%, 94.87%, 87.44%, 91.01%, 88.93%, and 95.59%, correspondingly.

**Table 4:** Comparative study of the SADDBN-AMOA approach^36]– [37^.

Approaches	Accu_y_	Prec_n_	Reca_l_	F1_score_
GBT	95.32	95.59	87.30	91.84
RT + LR	97.43	89.92	94.19	93.05
EM Clustering	97.64	96.78	92.29	94.87
DNN Algorithm	96.89	97.36	96.67	87.44
CNN-BiLSTM-GRU	89.48	93.21	96.37	91.01
Decision Tree	93.84	93.50	97.92	88.93
Conv-LSTM	97.62	87.88	92.63	95.59
SADDBN-AMOA	98.71	98.65	98.71	98.68

**Fig. 10:**
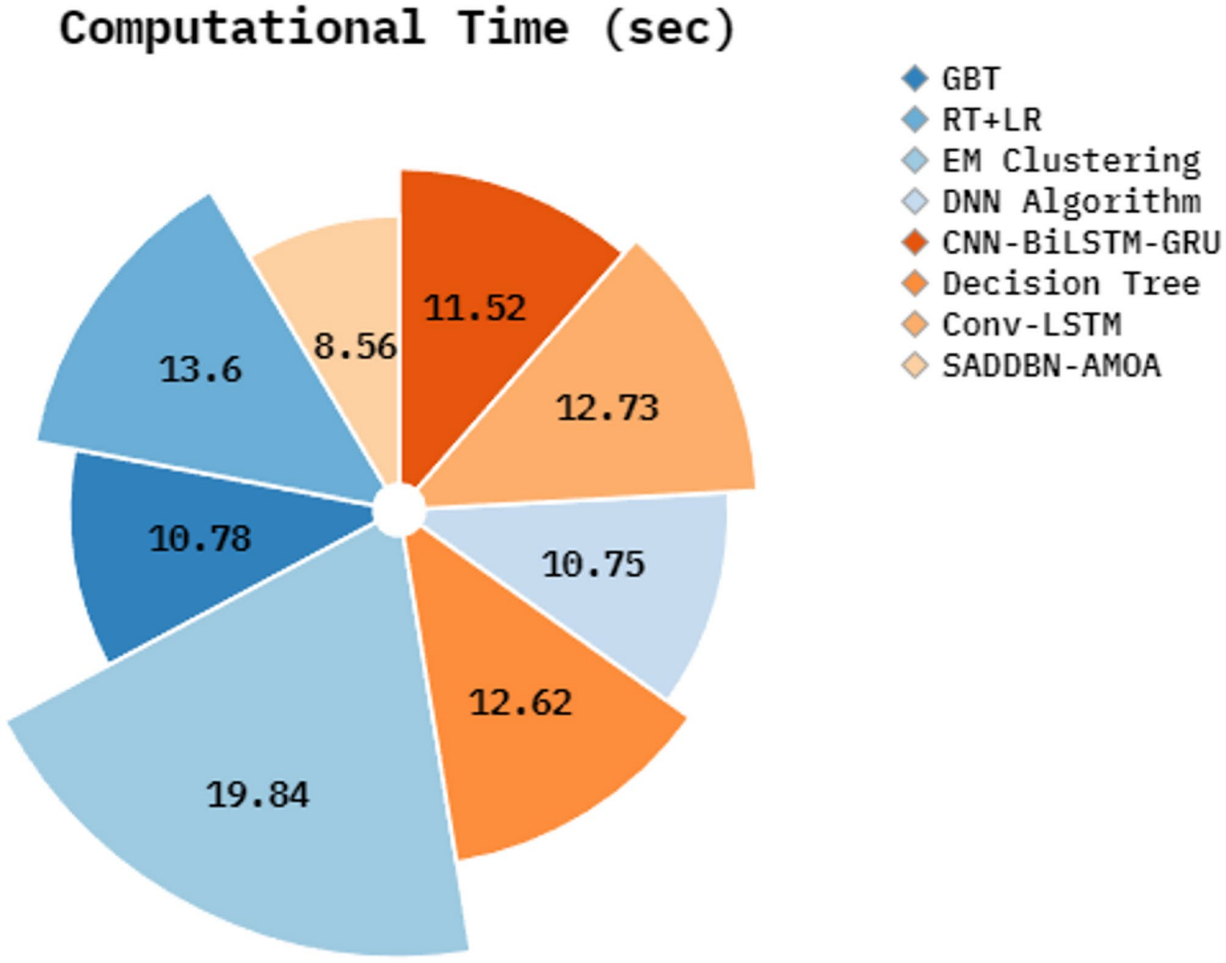
CT outcome of SADDBN-AMOA model with existing techniques.

Table 5; Fig. 10 establish the computational time (CT) of the SADDBN-AMOA methodology compared to existing techniques. Based on CT, the SADDBN-AMOA method provides a lesser value of 8.56 s, whereas the GBT, RT + LR, EM Clustering, DNN, CNN-BiLSTM-GRU, Decision Tree, and Conv-LSTM techniques attain greater CT of 10.78 s, 13.60 s, 19.84 s, 10.75 s, 11.52 s, 12.62 s, and 12.73 s, individually.

**Table 5:** CT analysis of the SADDBN-AMOA model with existing techniques.

Approaches	CT (sec)
GBT	10.78
RT + LR	13.60
EM Clustering	19.84
DNN Algorithm	10.75
CNN-BiLSTM-GRU	11.52
Decision Tree	12.62
Conv-LSTM	12.73
SADDBN-AMOA	8.56

## Conclusion

This manuscript proposes a novel SADDBN-AMOA technique in smart city-based IoHT networks. The main aim of the SADDBN-AMOA technique is to provide a resilient attack detection method in the IoHT environment of smart cities to mitigate security threats. The data pre-processing phase applies the Z-score normalization method for converting input data into a structured pattern. For the selection of feature process, the proposed SADDBN-AMOA model utilizes the SMO method to select the most related features from the data. Moreover, the DBN method is used for the attack classification method. Finally, the IHHO approach fine-tunes the hyperparameter values of the DBN method, leading to superior classification performances. The effectiveness of the SADDBN-AMOA method is investigated under the IoT healthcare security dataset. The experimental validation of the SADDBN-AMOA method illustrated a superior accuracy value of 98.71% over existing models. The limitations of the SADDBN-AMOA method comprise using a static dataset, which may not capture real-time discrepancies or unseen attack patterns in dynamic IoHT environments. The performance may also degrade when exposed to highly imbalanced or noisy data in real-world scenarios. Scalability remains a concern when deployed across large, distributed healthcare infrastructures with heterogeneous devices. Moreover, the model’s response under resource-constrained edge devices was not extensively examined. The reliance on pre-collected data limits adaptability to growing threat landscapes. Future enhancements include integration with real-time streaming data, lightweight model compression techniques for edge deployment, and adaptive mechanisms for handling concept drift. Collaborative validation across diverse healthcare setups will additionally strengthen reliability and generalizability.

## Data Availability

The data that support the findings of this study are openly available in Kaggle repository at https://www.kaggle.com/datasets/faisalmalik/iot-healthcare-security-dataset, reference number [35].

## References

[1] Aruna Santhi, J. & Vijaya Saradhi, T. Attack detection in medical internet of things using optimized deep learning: Enhanced security in healthcare sector. *Data Technol. Appl.* **55** (5), 682–714 (2021).

[2] Saheed, Y. K. & Arowolo, M. O. Efficient cyber-attack detection on the internet of medical things-smart environment based on deep recurrent neural network and machine learning algorithms. *IEEe Access.* **9**, 161546–161554 (2021).

[3] Al Abdulwahid, A. Detection of middlebox-based attacks in healthcare internet of things using multiple machine learning models. *Comput. Intell. Neurosci.* **2022**(1), 2037954 (2022). 36479020 10.1155/2022/2037954 PMC9722287

[4] Shankar, K. Improving the security and authentication of the cloud with IoT using hybrid optimization based quantum hash function. *J. Intell. Syst. Internet Things*. **1** (2), 61–61 (2021).

[5] Ravi, V., Pham, T. D. & Alazab, M. Deep learning-based network intrusion detection system for internet of medical things. *IEEE Internet Things Mag*. **6** (2), 50–54 (2023).

[6] Ahmad, S. et al. Deep learning enabled disease diagnosis for secure internet of medical things. *Comput. Mater. Contin.* **73**(1). (2022).

[7] Chaganti, R. et al. A particle swarm optimization and deep learning approach for intrusion detection system in internet of medical things. *Sustainability*, **14**(19), 12828. (2022).

[8] Si-Ahmed, A., Al-Garadi, M. A. & Boustia, N. Survey of machine learning based intrusion detection methods for internet of medical things. *Appl. Soft Comput.* **140**, 110227. (2023).

[9] Popoola, S. I., Adebisi, B., Hammoudeh, M., Gui, G. & Gacanin, H. Hybrid deep learning for botnet attack detection in the internet-of-things networks. *IEEE Internet Things J.* **8** (6), 4944–4956 (2020).

[10] Saini, P. Improved method for enhanced quality of service in IoHT task dependency optimization. *J. Cybersecur. Inform. Manag.*, **12**(2). (2023).

[11] Kumar, M., Singh, S. K. & Kim, S. Hybrid deep learning-based cyberthreat detection and IoMT data authentication model in smart healthcare. *Future Gener. Comput. Syst*. **166**, 107711. (2025).

[12] Amjath, M., Henna, S. & Rathnayake, U. Graph representation federated learning for malware detection in internet of health things. *Results Eng.* **25**, 103651 (2025).

[13] Benmalek, M., Seddiki, A. & Haouam, K. D. *SNN-IoMT: A Novel AI-Driven Model* (for Intrusion Detection in Internet of Medical Things, 2025).

[14] Syeda, Z. R. & Syed, R. S. Cyber attack detection for internet of health things through federated deep learning technique. In *2024 Annual Computer Security Applications Conference Workshops (ACSAC Workshops)* (pp. 194–200). IEEE. (2024).

[15] Nasayreh, A. et al. Automated detection of cyber attacks in healthcare systems: A novel scheme with advanced feature extraction and classification. *Comput. Secur*. **150**, 104288. (2025).

[16] Algethami, S. A. & Alshamrani, S. S. A deep learning-based framework for strengthening cybersecurity in internet of health things (IoHT) environments. *Appl. Sci.**14* (11), 4729. (2024).

[17] Albattah, A. & Rassam, M. A. Detection of adversarial attacks against the hybrid convolutional long short-term memory deep learning technique for healthcare monitoring applications. *Appl. Sci*. **13**(11), 6807. (2023).

[18] Bhowmik, T. & Banerjee, I. EEPPDA—Edge-enabled efficient privacy‐preserving data aggregation in smart healthcare internet of things network. *Int. J. Netw. Manag*. **33** (1), e2216 (2023).

[19] Saheed, Y. K. & Chukwuere, J. E. CPS-IIoT-P2Attention: Explainable privacy-preserving with scaled dot-product attention in cyber physical system-industrial IoT network. *IEEE Access* (2025).

[20] Gupta, K., Sharma, D. K., Gupta, K. D. & Kumar, A. A tree classifier based network intrusion detection model for Internet of Medical Things. *Comput. Electr. Eng.* **102**, 108158. (2022).

[21] Saheed, Y. K. & Misra, S. CPS-IoT-PPDNN: A new explainable privacy preserving DNN for resilient anomaly detection in cyber-physical systems-enabled IoT networks. *Chaos Solitons Fractals*, **191**, 115939. (2025).

[22] Gupta, K., Gupta, K. D., Kumar, D., Srivastava, G. & Sharma, D. K. Bids: Blockchain and intrusion detection system coalition for Securing internet of medical things networks. *IEEE J. Biomed. Health Informatics* (2023). 10.1109/JBHI.2023.3325964 37856270

[23] Khan, S., Imtiaz, N., Biswas, A. K., Bin Siddique, Z. & Khan, Q. A. An expert hybrid federated learning and trust management for security, efficiency, and power optimization in smart health systems. *IEEE Access*. (2025).

[24] Saheed, Y. K., Abdulganiyu, O. H. & Ait Tchakoucht, T. A novel hybrid ensemble learning for anomaly detection in industrial sensor networks and SCADA systems for smart city infrastructures. *J. King Saud Univ.-Comput. Inf. Sci.* **35**(5), 101532. (2023).

[25] Darabkh, K. A. & Al-Akhras, M. Towards optimized IoT sensor networks for smart cities: Centrality-Aware Position-Based Occlusion-Driven and role dynamics solutions for clustering and routing. *IEEE Internet Things J.* (2025).

[26] Saheed, Y. K., Abdulganiyu, O. H., Majikumna, K. U., Mustapha, M. & Workneh, A. D. ResNet50-1D-CNN: A new lightweight resNet50-One-dimensional convolution neural network transfer learning-based approach for improved intrusion detection in cyber-physical systems. *International Journal of Critical Infrastructure Protection*, *45*, p.100674. (2024).

[27] Dandamudi, S. R. P., Sajja, J. & Khanna, A. Leveraging artificial intelligence for data networking and cybersecurity in the united States. *Int. J. Innov. Res. Comput. Sci. Technol.* **13**, 34–41 (2025).

[28] Saheed, Y. K. & Chukwuere, J. E. Xaiensembletl-iov: A new explainable artificial intelligence ensemble transfer learning for zero-day botnet attack detection in the internet of vehicles. *Results Eng.*, **24**, 103171. (2024).

[29] Manivannan, R. & Senthilkumar, S. Intrusion detection system for network security using novel adaptive recurrent neural network-based fox optimizer concept. *Int. J. Comput. Intell. Syst.*, **18**(1), 37. (2025).

[30] Saheed, Y. K., Misra, S. & Chockalingam, S. May. Autoencoder via DCNN and LSTM models for intrusion detection in industrial control systems of critical infrastructures. In *2023 IEEE/ACM 4th International Workshop on Engineering and Cybersecurity of Critical Systems (EnCyCriS)* (pp. 9–16). IEEE. (2023).

[31] Wang, X., Yang, X., Zhou, J. & Ren, H. Z-Score‐based improved TOPSIS method and its implementation for elderly people health examination results evaluation: A statistic case study in Harbin, China. *Health Soc. Care Commun.*, **2025**(1), 5974609. (2025).

[32] Mariappan, Y., Ramasamy, K. & Velusamy, D. An optimized deep learning based hybrid model for prediction of daily average global solar irradiance using CNN SLSTM architecture. *Sci. Rep.* **15**(1), 10761. (2025). 10.1038/s41598-025-95118-3 PMC11953468 40155655

[33] Hossain, Y., Ferdous, Z., Wahid, T., Rahman, M. T., Islam, M. A. & Dey, U. K. and Enhancing intrusion detection systems: Innovative deep learning approaches using CNN, RNN, DBN and autoencoders for robust network security. *Appl. Comput. Sci.* **21** (1), 111–125 (2025).

[34] Wang, J. et al. A deep hybrid prediction framework for Building operational carbon emissions: Integrating enhanced extreme learning machines. *Energy Rep.* **13**, 4126–4140 (2025).

[35] https://www.kaggle.com/datasets/faisalmalik/iot-healthcare-security-dataset

[36] Varadharajan, V., Tupakula, U. & Karmakar, K. Secure monitoring of patients with wandering behavior in hospital environments. *IEEE Access.* **6**, 11523–11533 (2017).

[37] Akhi, M., Eising, C. & Dhirani, L. L. TCN-Based DDoS detection and mitigation in 5G Healthcare-IoT: A frequency monitoring and dynamic threshold approach. *IEEE Access* (2025).

